# The Nobel Prize in Physiology or Medicine—2020

**DOI:** 10.1007/s11224-021-01731-z

**Published:** 2021-02-09

**Authors:** Krisztina Hagymási

**Affiliations:** 1st Department of Surgery and Interventional Gastroenterology, Semmelweis University, Budapest, Hungary

**Keywords:** Nobel Prize, Hepatitis C virus, Screening, Direct-acting antiviral agents, Elimination

## Abstract

At the time of COVID-19 coronavirus pandemia, the Nobel Prize of Physiology or Medicine 2020 was awarded jointly to three researchers Harvey J. Alter, Charles M. Rice, and Michael Houghton for the discovery of Hepatitis C virus. Their works contributed to the isolation of the blood-borne virus, causing chronic hepatitis in 80% of infected person, resulting in cirrhosis, and in elevated risk of liver failure and hepatocellular carcinoma formation. Their results created the basis of HCV screening of blood, and blood products, achieving more than 95% cure of infected people without nearly side effects with direct-acting antiviral agents, supporting the goal of the WHO targeting the elimination of viral hepatitis by 2030.

## Nobel recognition

The Nobel Prize 2020 in Physiology or Medicine has been awarded jointly to three researchers: Harvey J. Alter, Charles M. Rice, and Michael Houghton for the discovery of Hepatitis C virus.

Harvey J. Alter (US National Institutes of Health) co-discovered the Australia antigen with B. Blumberg and later with his co-workers defined a blood-borne “non A non B” hepatitis virus responsible for hepatitis acquired by blood or blood product transfusion in the middle of the 1970s.

Michael Houghton (University of Alberta, Canada) also co-discovered the Hepatitis D genome in 1986, with his colleagues isolated a clone derived from a novel RNA virus belonging to the Flavivirus family in infected chimpanzees, and it was named Hepatitis C virus in 1989.

Charles M. Rice and co-workers (Rockefeller University, NY) cultured the first infectious Hepatitis C clone for use in studies on chimpanzees. His team showed that a strain of an acute form of the virus identified in a human patient can be forced to replicate in a laboratory setting. They described the whole HCV genome in 1996 and provided evidence for its infective nature in the next year. Their other key results were important (f.e. description of many proteins required for viral entry) in the screening of blood products and development of active antiviral agents (https://www.nobelprize.org/prizes/medicine/2020/summary/).

## Background

Europe has the largest burden of liver disease in the world. The significance of liver disease continues to grow in Europe. Liver cirrhosis is responsible for more than 1 million deaths annually worldwide, and the majority of these deaths is preventable. The nonalcoholic fatty liver (steatosis/steatohepatitis) disease developing in connection with metabolic syndrome and obesity, alcohol consumption, viral hepatitis (Hepatitis B, C, D), autoimmune liver disorders, and storage disorders (Wilson’s disease, hemochromatosis, α1-antitrypsine deficiency) are is the main chronic liver disorder. Regarding mortality, viral hepatitis remains a high priority throughout Europe [1, 2].

B. Blumberg (US NIH) identified that one form of blood-borne hepatitis was caused by a virus that became known as Hepatitis B virus in the 1960s. The discovery led to the development of diagnostic tests and an effective vaccine to prevent spread by blood donation. Blumberg was awarded the Nobel Prize in Physiology or Medicine in 1976 for this discovery (https://www.nobelprize.org/prizes/medicine/1976/blumberg/facts/).

### Hepatitis C virus

Hepatitis C virus is another significant etiological factor of chronic viral hepatitis. It is a positive single-stranded RNA virus with a small membrane and belongs to the Flaviviridae family and the only member of the *Hepacivirus* genus. The viral genome is composed of approximately 9600 nucleotides and encodes a single-open reading frame of 3010 amino acids. There are 7 major genotypes which are differed from each other by ~ 25–35% at nucleotide level and 67 subtypes which differ from each other by ~ 15–25% at the nucleotide level. The genotype 1 is the most frequent globally [3, 4].

**Fig. 1:**
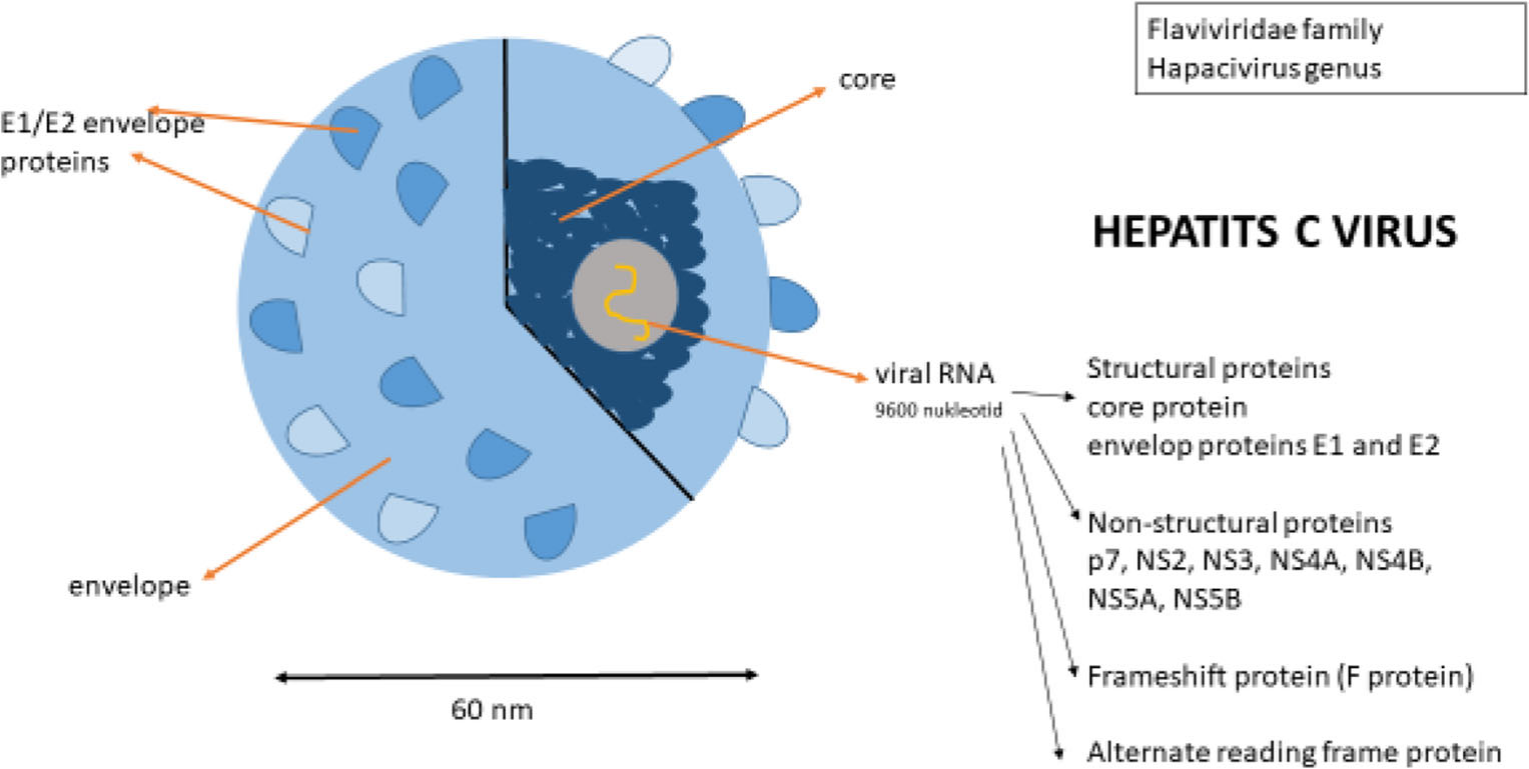
The Hepatitis C virus

It is important to understand the main stages of the HCV viral cycle in order to understand the mode of action of different treatments. However, the exact molecular mechanisms underlying this cycle are not completely understood and remain extremely complex. The virus has two surface glycoproteins E1 and E2, crucial in the clathrin-mediated endocytosis of HCV. The virus enters cells by interacting with membrane molecules on the basolateral hepatocyte membrane. It binds to CD81 surface receptors found on hepatocytes and B lymphocytes. Other molecules that seem to be targets to gain entry are LDL receptor, claudin-1, occludin, and scavenger receptor B type 1. It can coat itself in LDL and VLDL using their receptors on hepatocytes to gain entrance and avoid neutralizing antibodies in the serum. After disruption of the viral capsid in the endocyte and being released into the cytosol, the positive RNA strand is translated at the rough endoplasmic reticulum. The primary translation product is ~ 3000 amino acid long polyprotein precursor which contains structural and non-structural proteins of HCV. Then, the polyprotein is cleaved by host and viral proteases into three structural proteins (core protein, envelop proteins E1 and E2) as well as seven non-structural proteins (p7, NS2, NS3, NS4A, NS4B, NS5A, NS5B) of the viral replication machinery and in addition a frameshift protein (F protein) or alternate reading frame protein (ARFP). NS2 is important in virus assembly and release. NS3 and NS4A form a protease complex responsible for cleaving parts of the polyprotein. Virus-host interaction is mediated by the membrane-associated protein NS4B. NS5A is involved in replication. The key enzyme of HCV RNA replication is NS5B, which is an RNA-dependent RNA polymerase. With NS3 helicase, it synthesizes a minus-strand RNA that serves as template for numerous plus-stranded RNA. The precise mechanism of viral assembly and release is not well understood, but it is believed that it is a multistep procedure involving several cellular factors and most of the viral components [4–6] (Fig. 1).

### Epidemiology and clinical signs

HCV causes 3–4 million new infections and 366,000 deaths worldwide. Approximately 80% of infected person develops chronic liver injury and chronic hepatitis during decades resulting in liver cirrhosis and hepatocellular carcinoma formation. It is responsible for the 27% of liver cirrhosis and 25–31% of primary liver cancer. Estimated worldwide frequency is 1–3%, with 70 million chronically infected people in 2017; fewer than 20% of those living with HCV are aware of their infection. There is no vaccine to prevent the infection [4–7].

The main route of infection is via infected blood; blood product transfusion has been changed after the discovery of the virus and screening of blood products (1992). Nowadays, the main risk groups are i.v. drug users and special subgroups of sexuality (men who have sex with men).

**Fig. 2:**
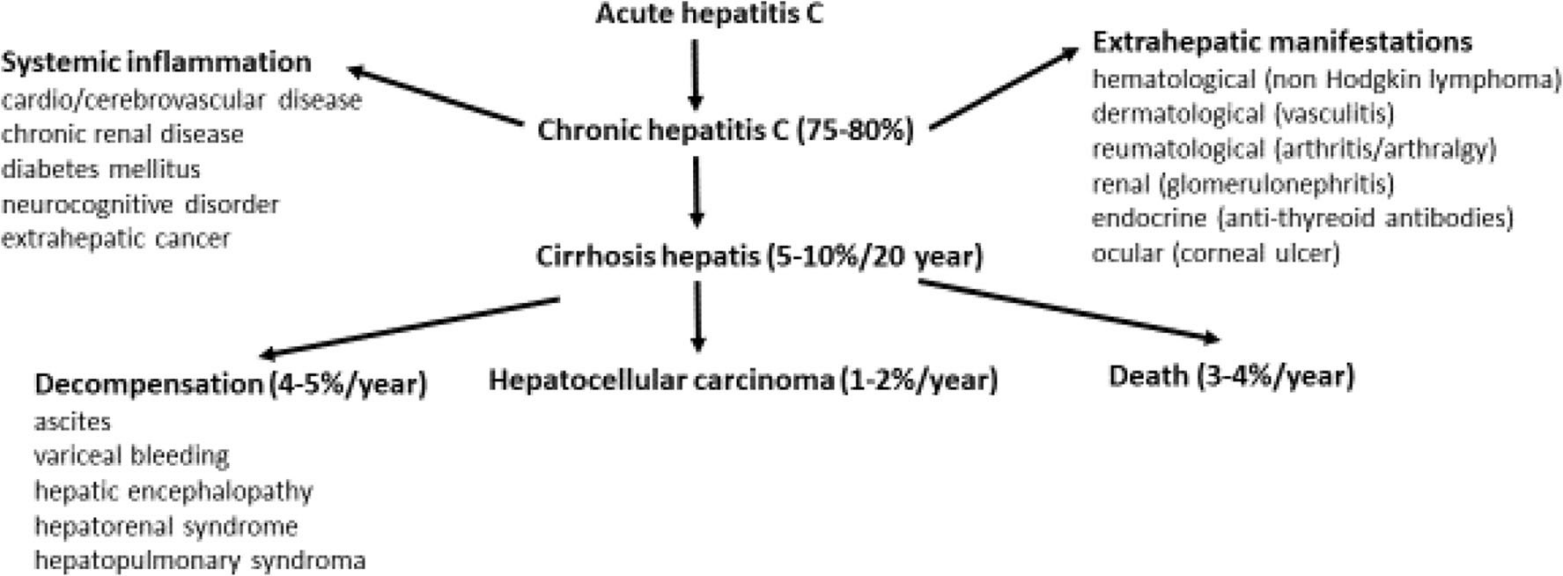
Pathophysiology of Hepatitis C virus infection

After a 15–120-day incubation period, symptoms of acute hepatitis develop in about 30% of infected people, more than 80% develop chronic hepatitis, later 10–20% of patients have cirrhosis, and 1–4% has liver cancer and hepatocellular carcinoma. HCV infection is not only a hepatic infection, and at least one extrahepatic finding (cutaneous involvement, rheumatologic, hematological, renal, neurological manifestations) is seen in 40–74% of HCV-infected patients. The chronic infection is also responsible for chronic systemic inflammation that will lead to insulin resistance with a 1.5 times higher risk of diabetes, 2–3 times higher risk of cardio-, cerebro-, or renovascular diseases, and an increased risk of extrahepatic cancers [5] (Fig. 2).

### Therapy

The interferon alpha was the first approved drug by the FDA and used in patients with Hepatitis C infection in 1991, but the treatment success rate was only 10%. In 1998, co-therapy with ribavirin, which can inhibit RNA virus synthesis, increased the therapeutic effect by 30–50%.

In 2001, the development of pegylated interferon has achieved long-term therapeutic effects. Forty-eight-week combined treatment with nucleoside analogue ribavirin further increased the therapeutic effect to 40–80%, depending on the genotype. Until recently, the combination treatment of peginterferon/ribavirin was the most widely used treatment with poor clinical tolerance, with many side effects (flu-like syndrome, neurocognitive disorders, myelosuppression with neutropenia, thrombocytopenia, and hemolytic anemia). Therapeutic efficacy was limited by several factors: extensive fibrosis, overweight, genotype 1, HIV-associated infection, or insulin resistance [5].

**Fig. 3:**
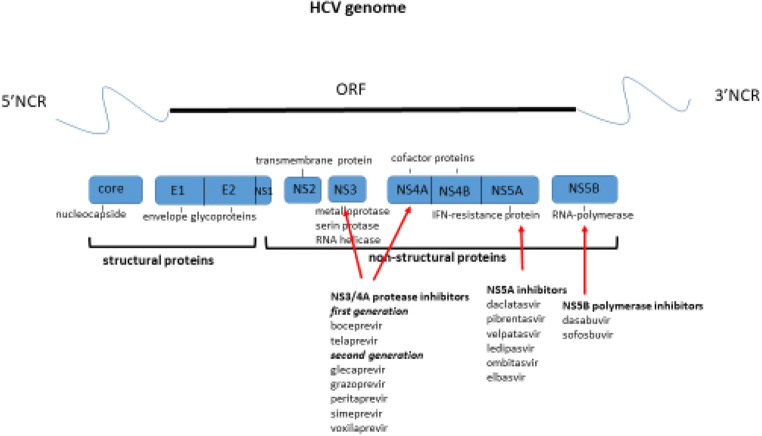
Direct-acting antiviral agents

Availability of direct oral antiviral agents and specific inhibitors of viral proteins has been a real therapeutic revolution. Direct-acting antiviral agents (DAAs) act directly on specific sites in HCV non-structural proteins to block HCV replication. They are classified into NS3/4A protease inhibitors, NS5A inhibitors, and NS5B polymerase inhibitors. The first protease inhibitors, telaprevir and boceprevir, used from 2011 to 2014, were combined with standard treatment with pegylated interferon and ribavirin: they allowed having of a 24-week treatment duration of genotypes 1 and 4 and cured about 70–80% of patients, but the safety issues remained. In 2013, the NS5B polymerase inhibitor (sofosbuvir) was approved by the FDA, so the interferon-free era has been started. Since 2014, the interferon-free combination of 2–3 antivirals (protease inhibitors, paritaprevir, grazoprevir, glecaprevir, and voxilaprevir; polymerase inhibitors, sofosbuvir and dasabuvir; NS5A inhibitors, pibrentasvir, velpatasvir, ledipasvir, ombitasvir, elbasvir, and grazoprevir) has been used to cure almost all patients (sustained viral response more than 95%) for 8–12 weeks without side effects. The only limits that cannot be avoided today are the significant drug-drug interactions [5, 8, 9] (Fig. 3).

Virologic cure is usually accompanied by clinical improvement or even clinical cure of liver and extrahepatic manifestations [5].

### Elimination of viral hepatitis by 2030

HCV infection is the success of the translational research. From the discovery of the virus (1989), through the empiric interferon therapy, via exploring of viral life cycle and development of serological and non-invasive assessment of fibrosis by blood markers and morphological tests, from 2013, the direct-acting antiviral agents are used without interferon, without side effects with more than 95% efficacy. The history of chronic HCV therapy ends of the combination of pangenotypic tablets for 8–12 weeks [5].

The discovery of HCV and development of interferon-free therapy is one of main success in the medicine in the past decades, creating a chance to stop the increasing burden of HCV infection, as well as decreasing mortality. The HCV infection is the only chronic viral infection which can be cured: so the so-called sustained viral response can be achieved, defined by the undetectable HCV-RNA after the end of therapy with 12 (or 24) weeks [5].

In 2016 the 194 member states agreed on a global hepatitis strategy to eliminate viral hepatitis by 2030 set as a goal by the WHO. Elimination was defined as 90% reduction in hepatitis (HBV and HCV) incidence and 65% reduction in mortality. To achieve the WHO targets, treatment would need to be increased from 150,000 patients annually using DAAs at 95% SVR in 2015 to 187,000 in 2025, with expansion of treatment age to 15–74 years old, and treatment of all fibrosis stages [2].

The direct-acting antiviral drug research, targeting viral replication, is ongoing with the possibility of new types of antiviral drugs that target host factors that interfere and is essential for the virus (viral entry inhibitors-antagonists-peptides and antibodies targeting E2-CD81, E2-SR-B1 interactions, ApoE, E1CD81-CLDN1 co-receptor complex formation, viral trafficking and internalization, or viral fusion via interference with E1 or targeting of lipids and membrane fluidity, miRNA-122 inhibitors, cyclophilin inhibitors). Increased accurate, simple, rapid testing and harm reduction through needle-exchange and opioid-substitution therapy are likely to reduce the burden of viral hepatitis infection in both general and high-risk population groups. Increasing knowledge about viral cycling could provide further information to develop an effective vaccine, which is a challenge that still remains to be solved [2, 4, 6].

## Summary

The results of awarded researchers created the basic of HCV elimination, which is one of the most frequent chronic viral hepatitis. It is the only curable chronic viral hepatitis. The elimination can be finalized by the screening of high-risk groups and development of national screening program.
